# Benign ferroelastic twin boundaries in halide perovskites for charge carrier transport and recombination

**DOI:** 10.1038/s41467-020-16075-1

**Published:** 2020-05-05

**Authors:** Xun Xiao, Wenhao Li, Yanjun Fang, Ye Liu, Yuchuan Shao, Shuang Yang, Jingjing Zhao, Xuezeng Dai, Rashid Zia, Jinsong Huang

**Affiliations:** 1 Department of Applied Physical Sciences, University of North Carolina, Chapel Hill, NC 27599 USA; 2 School of Engineering and Department of Physics, Brown University, Providence, RI 02912 USA; 3 Department of Mechanical and Materials Engineering and Nebraska Center for Materials and Nanoscience, University of Nebraska-Lincoln, Lincoln, NE 68588 USA

**Keywords:** Energy science and technology, Materials science

## Abstract

Grain boundaries have been established to impact charge transport, recombination and thus the power conversion efficiency of metal halide perovskite thin film solar cells. As a special category of grain boundaries, ferroelastic twin boundaries have been recently discovered to exist in both CH_3_NH_3_PbI_3_ thin films and single crystals. However, their impact on the carrier transport and recombination in perovskites remains unexplored. Here, using the scanning photocurrent microscopy, we find that twin boundaries have negligible influence on the carrier transport across them. Photoluminescence (PL) imaging and the spatial-resolved PL intensity and lifetime scanning confirm the electronically benign nature of the twin boundaries, in striking contrast to regular grain boundaries which block the carrier transport and behave as the non-radiative recombination centers. Finally, the twin-boundary areas are found still easier to degrade than grain interior.

## Abstract (web summary)

Grain boundaries are known to be generally detrimental to the operation of metal halide perovskite solar cells. Here Xiao et al. show with scanning photocurrent microscopy that ferroelastic twin boundaries are benign to the electrical properties in crystalline samples, in contrast to grain boundaries.

## Introduction

Metal halide perovskite (MHP) materials have exhibited great potential in realizing next-generation low cost and high efficiency solar cells^1–10^, and their certified power conversion efficiency (PCE) has already exceeded 24% for small area devices^11^ and 16.4% for minimodules with area larger than 60 cm^2^^12^. The rapid increase of PCE in MHP solar cells during the past few years relies largely on the grain boundary (GB) engineering of the polycrystalline thin film, including reducing the GB density with increased grain size^8,13–15^ and passivating the GBs^16–18^, because GBs in perovskites are known as locations with higher charge recombination rate. In addition to GBs, domain like structure inside the grains of CH_3_NH_3_PbI_3_ (MAPbI_3_) thin films was firstly discovered by Hermes et al. with piezoresponse force microscopy (PFM)^19^. Ferroelectric polarization was proposed to be responsible for these domains under PFM characterization^20^. Rohm et al. demonstrated these domains are ferroelectric by observing domain walls generation and extinction with applied electric poling^21^. Apart from that, Wei et al. reported polarization-electric filed hysteresis in perovskite layer, which provided strong support for ferroelectricity in perovskite^22,23^. Breternitz et al. further proved the atom displacement in the crystal lattice and hence polarity of the unit cell of MAPbI_3_, which is a very important step to verify the ferroelectricity of this material. On the other hand, Gomez et al. claimed these domains in PFM are actually ferroelectricity-free by developing direct piezoelectric force microscopy (DPFM) to avoid involving artefacts that might be mistaken as ferroelectric signal in classical PFM, especially for the potential artefacts induced by ion migration, topography crosstalk or charged ions contamination^24^. In addition, the polarity of MAPbI_3_, one of essential conditions for ferroelectricity, has also been questioned^25^. While the ferroelectric nature is still under hot debate, the existence of ferroelasticity, or mobile twin boundaries under stress, in MAPbI_3_ has been well established. Strelcov et al. confirmed the ferroelastic nature of these domains based on the observation of the twin boundary (TB) motion under external stress in both MAPbI_3_ thin films and single crystals^26^. Meanwhile, using low-dose transmission electron microscopy (TEM) and selected area electron diffraction (SAED), Rothmann et al. directly observed the structure of these ferroelastic twin-domains, and identified that they are formed as a result of the cubic-to-tetragonal phase transition of MAPbI_3_^27^.

The formation of TBs in MHPs is generally driven by the strain in perovskite films which in return release the strain in the material. For MHP thin films, the strain could be induced by lattice thermal expansion coefficient mismatching during annealing process. The presence of large strain in all polycrystalline perovskites was observed in eventually all type of perovskite films made by different methods, as long as thermal annealing is needed^28^. For flexible perovskite solar cells, additional strain is induced when the devices are under-bending working status. The strain in the polycrystalline films has been shown to accelerate the degradation of MHP films^28^, thus releasing strain by the formation of TBs is expected to enhance the stability of flexible perovskite devices and making the ceramic like MHPs to be more flexible. Apart from impacts on stability, whether TBs would affect the MHP device efficiency is still an open question.

However, the impact of the TBs on MHP device operation, especially on charge transport, remains largely unexplored. TBs are two-dimensional defects, like grain boundaries, but is a special type of 2D defect with no dangling bonds. It is still unknown whether TBs would behave like GBs, which has been frequently reported to cause non-radiative charge recombination or blocking carrier transport and thus decrease the PCE of perovskite solar cells^16^. And it is not known yet whether TBs would act as vulnerable sites for external stimuli to induce faster degradation. Hence, understanding the impact of TBs on the charge transport and stability of MHP materials would be of great interests for further increasing the PCE and stability of MHP solar cells.

Here, we use MHP single crystals with TBs and multi-crystalline films with GBs as a control system to study the impact of ferroelastic TBs on carrier transport. Using scanning photocurrent microscopy (SPCM) and the photoluminescence (PL) imaging techniques, the electronically benign nature of the TBs has been revealed. Our experimental results indicate that TBs affect neither the carrier transport when carrier transport across them, nor behave as non-radiative recombination centers, in striking contrast to the regular GBs in MHP.

## Results

### Thin single crystal with ferroelastic twin boundaries

**Fig. 1:**
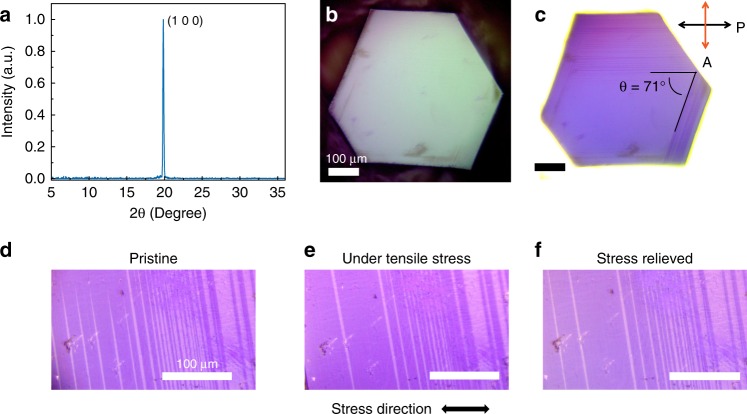
Ferroelastic domain pattern observed by polarized optical microscope. **a** X-ray diffraction pattern of the as-grown CH_3_NH_3_PbI_3_ (MAPbI_3_) single crystals. **b** and **c** Regular (**b**) and polarized optical microscope (**c**) images of the MAPbI_3_ single crystal; The two groups of domain patters intercept at about 71^o^ in the top (100) plane. **d** to **f** Polarized light optical images of domain pattern in MAPbI_3_ single crystal before the application of external stress (**d**), under tensile stress (**e**), and after reliving the stress (**f**); The arrow in **e** indicates the applied stress direction. The scale bars are 100 μm.

The MHP single crystals were grown on glass substrates by a recently developed space confined lateral crystal growth method^29,30^, which produces thin single crystals with typical lateral dimensions of 0.5 to 5 mm and thicknesses of 10 to 50 μm. Solar cells and photodetectors fabricated with these single crystals as photoactive layer already show a high PCE over 21%^31^ and an excellent sensitivity with low light detection limit of 0.35 pW cm^−2^ ^30^, demonstrating the high quality of the single crystals grown by this method. The single sharp diffraction peak in the XRD pattern shown in Fig. 1(a), which is assigned to (100) plane of MAPbI_3_, confirms the single crystal nature of the samples. In principle, the ferroelastic twin domains should have different orientation and hence result in the splited diffraction peaks, as observed in reciprocal space like the SAED in TEM results^27^. However, for the theta-2theta XRD scan, it’s only sensitive to the d-spacing of the planes that are parallel to the substrate rather than reciprocal angles of the twin domains. And thus, the split diffraction peaks could not be observed in this XRD spectra. As shown in Fig. 1(b), the as-grown MAPbI_3_ single crystal exhibits a hexagon shape. The ferroelastic twin domains with stripe contrast in the as-grown MAPbI_3_ single crystals can be clearly resolved through the polarized optical microscope under the reflection mode using the crossed Nicols configuration (Fig. 1(c)), which is a simple yet effective method to differentiate domains of different crystallographic orientations. To confirm the ferroelastic property of the domains, we first transferred the single crystals from the glass substrate to a flexible polyethylene terephthalate substrate. We then applied stress to the substrate to examine the ferroelasticity with the strain induced domain wall motion. As shown in Fig. 1(d–f), the stripe pattern changed under the external tensile stress, verifying the ferroelastic nature of these domains. Another important observation is that some domain boundaries did not completely return to the original location after the applied strain was released, which corresponds to a hysteresis behavior for the motion of these ferroelastic domains. It is noted that two groups of domain stripes intersect with an angel of around 71° shown on the top (100) surface, agreeing with previous PFM observations at grain level. Previous TEM and SAED studies concluded that the TBs in MAPbI_3_ thin films are {112}_t_ planes (the subscript ‘t’ denotes the tetragonal phase)^27^. Based on the geometry, it could be derived that the only low-index planes that can give an intersection angle of 71° are the {112}_t_ group on the (100) planes, further confirming the mirror planes of the ferroelastic domains in MAPbI_3_ single crystals are {112}_t_ planes.

### Carrier transport through TBs study with SPCM

**Fig. 2:**
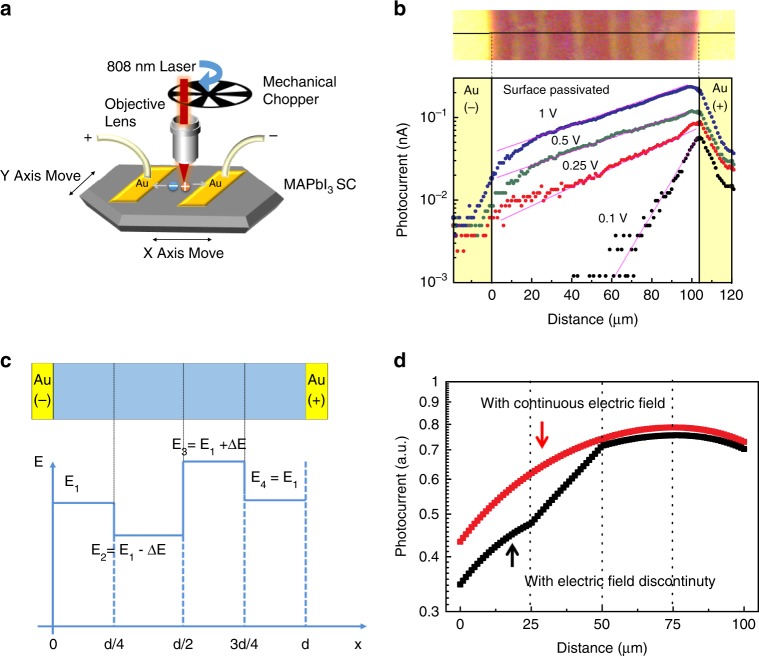
Carrier transport probed by scanning photocurrent microscope (SPCM). **a** Schematic illustration of the SPCM setup. **b** Polarized optical image of the MAPbI_3_ single crystal lateral device (upper panel) and the photocurrent line scan profile across the conductive channel (lower panel) with increased electric field from 1 V mm^−1^ to 10 V mm^−1^ after crystal surface passivation. The horizontal black solid line indicates the photocurrent line-scan trace, the pink solid line is a guide to eye. **c** Proposed electric field distribution across the conductive channel. **d** The corresponding simulation results of the photocurrent profile without and with the electric field discontinuity.

The impact of the ferroelastic TBs on the carrier transport crossing them is investigated with SPCM which has been frequently used to study the carrier diffusion length in MHP and other optoelectronic materials^32–35^. The schematic of our measurement setup is shown in Fig. 2a. The Au electrodes were thermally evaporated on the single crystal surface through a shadow mask with the channel width of about 100 μm. The single crystal was then fixed on a piezomotor driven *X*–*Y* scanning stage, and a laser beam was first modulated by a mechanical chopper and then focused onto the sample with a spot size of approximately 2 μm. During the measurement, the sample on the *X*–*Y* stage moved along the *X* or *Y* axis with a step of 1 μm, while the photocurrent signal was recorded by a lock-in amplifier. The samples were excited by a laser with wavelength of 808 nm which has a penetration depth of 3.3 μm. This allows us to visualize the photocurrent signal coming from the carriers that transport across TBs in the bulk crystal, which minimizes the impact of the surface charge recombination and highlights the impact of the TBs on carrier transport. The cross-section view of the measurement has been sketched as Supplementary Fig. 1. In theory, the photocurrent profile within the conductive channel under external bias can be calculated based on a modified Hecht equation, which has been widely used in the radiation detector field:

1 $$I(x) = \frac{{\eta qP}}{{h\nu d}}\left( {\mu _{\mathrm{e}}\mathop {\smallint }\nolimits_{\!\!\!0}^{t_{\mathrm{e}}} e^{ - {\textstyle{{t^{\prime}} \over \tau }}}E\left( {x_{\mathrm{e}}(t^{\prime})} \right){\mathrm{d}}t^{\prime} + \mu _{\mathrm{h}}\mathop {\smallint }\nolimits_{\!\!\!0}^{t_{\mathrm{h}}} e^{ - {\textstyle{{t^{\prime}} \over \tau }}}E\left( {x_{\mathrm{h}}(t^{\prime})} \right){\mathrm{d}}t^{\prime}} \right)$$

where *q* is the elementary charge, *η* is the external quantum efficiency, *P* is the incident light intensity, *h* is the Planck constant, *ν* is the incident light frequency, *d* is the conductive channel width, *μ*_e_ and *μ*_h_ are the electron and hole mobilities, respectively, *τ* is the carrier recombination lifetime, *t*_e_ and *t*_h_ are the electron and hole drift times, respectively, *x*_e_(*t*) and *x*_h_(*t*) are the position of the electrons and holes with the drift time of *t* after generation, respectively, and *E*(*x*(*t*)) is the electric field intensity at the position *x*(*t*)^36^.

We selected some TBs whose intersection with top surface are in parallel with the electrode bars to do the SPCM measurements, as shown in the upper panel of Fig. 2b, since they are ideal to investigate the effect of ferroelastic TBs on the carrier transport across them. To further reduce the impact of charge recombination and ion migration at the crystal surface, we converted the surface of the MAPbI_3_ single crystals into wide bandgap, water-insoluble PbSO_4_ through the reaction of MAPbI_3_ with sulfate ions. It has been proved by us to be an effective method to dramatically reduce the surface defect density and enhance the surface stability in moisture environment^37^. The suppressed signal from surface current would further highlight the current signal from carriers that travel through the bulk of the crystal, which provides an ideal platform to study how the carriers transport across the TBs. As shown in Fig. 2b, the line-scan photocurrent profiles under an excitation power of 31.8 mW cm^−2^ which induces a carrier density of 1.3 × 10^13^ cm^−3^ and external fields from 1 V mm^−1^ to 10 V mm^−1^. The photocurrent peak position is closer to the anode, indicating a larger hole mobility than electron mobility in this sample, which is in accordance with previous results^38^. Notably, if the twin boundaries are charged and not fully compensated by free carriers, it would induce a discontinuity of the electric field across the TBs and thus influence the carrier transport^39^. To verify this, a model of SPCMs with non-uniform and uniform electric field have been established (Fig. 2c). Figure 2d shows inflection points would appear in the photocurrent profile at the electric field discontinuity position comparing with continuous photocurrent decay profile under uniform electric field. However, the absence of such inflection points at the TBs in the surface passivated samples indicates no electric field discontinuity at twin boundaries and TBs do not affect carrier transport across them. To study the impact of TBs on MAPbI_3_ stability, non-encapsulated MAPbI_3_ crystals with TBs were exposed to the ambient for 3 days with a relative humidity of 40%, and then SPCM was conducted. As shown in Supplementary Fig. 2, many inflection points appeared at the locations of TBs for the un-passivated crystals. It shows that the TBs are much easier to be attacked by moisture.

Enhanced conductivity at ferroelectric domain boundaries in oxide perovskite has been well studied, and these unique properties at domain boundaries are usually associated with atomic re-arrangement or change of chemistry of ions/atoms at the boundaries^40^. Previous study of atomic structure of TBs in MAPbI_3_ by high resolution TEM did not show notable difference of ions/atoms re-arrangement, which differed from domain boundaries in oxide perovskite and did not suggest a change of conductivity at TB^27^. Nevertheless, conductive atomic force microscopy (c-AFM) was carried out to directly verify the local conductivity at TBs with nanoscale resolution. The thin single crystal sample with thickness of around 10 μm was synthesized as mentioned before. The ITO substrates were used to grow the crystal and working as the bottom electrode. The c-AFM measurement setup was shown as Supplementary Fig. 3(a). The crystal sample was first polished to remove the surface contamination. Then it was analyzed under the polarized optical microscope to identify the TBs location. Twin boundaries were clearly observed, as shown in Supplementary Fig. 3(b). The morphology had been acquired by AFM measurement. As shown in Supplementary Fig. 3(c), the polished crystal is relatively flat while with some scratches were observed caused by the polish process. These scratches do not affect the electric measurement, since it is much smaller than the total thickness of the crystals. And the c-AFM mapping results in Supplementary Fig. 3(d) reveals that there is no obvious conductivity change at TBs location.

### Carrier diffusion imaged by PL imaging method

**Fig. 3:**
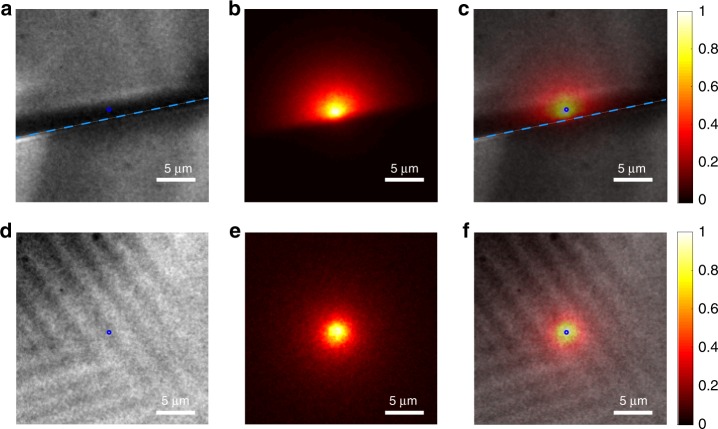
Capture carrier transport with photoluminescence (PL) imaging. **a** Polarized optical microscope image, (**b**) PL intensity image and (**c**) composite image of a MAPbI_3_ multi-crystal with GBs. The blue dashed line indicates the position of GB. **d** Polarized optical microscope image, (**e**) PL intensity image and (**f**) composite image of a MAPbI_3_ single crystal with TBs. The PL excitation position is indicated by the blue circle.

**Fig. 4:**
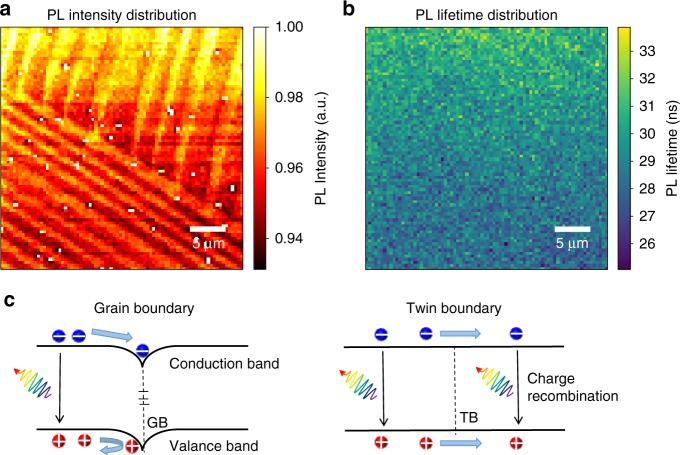
Charge carrier recombination dynamics at TBs and GBs. **a** and **b** Confocal PL intensity and lifetime mapping of TBs; (**c**) The proposed energetic diagram of MAPbI_3_ with GBs (left panel) or TBs (right panel) exhibiting the origin of their different impacts on charge carrier transport and recombination.

Apart from SPCM, PL imaging measurement was carried out to further confirm the benign nature of the TBs on carrier transport in MHP materials^41,42^. In this measurement, a 532 nm CW laser was focused on the sample to generate charge carriers in a small spot with a diameter of 250 nm which is near the diffraction limit of the excitation laser. The photoexcited electrons and holes diffuse in all directions in the sample, driven by carrier concentration gradient. During the diffusion process of the charge carriers, the radiative recombination of excess electrons and holes generates PL and the PL intensity reflects the carrier concentration at the location. Since PL emission needs the presence of both excess electrons and holes, the PL images would show a discontinuity at the boundary if either electrons or holes are blocked by the boundary. Therefore, the carrier diffusion process can be directly imaged based on the PL intensity distribution. We first performed this measurement on a multi-crystalline MAPbI_3_ sample with grain boundaries to find out the effect of regular GBs on the carrier transport. A sample with a grain size significantly larger than the light excitation spot size is needed for this measurement. This sample was grown by spreading the 1.5 M MAPbI_3_ GBL solution onto the hydrophobic material treated cover glass and then slowly solvent evaporation at 100 °C. This method produced large grains with lateral sizes from 0.1 to 1 mm, while the GBs were formed at the contact of two adjacent crystals. The GB position is indicated by the blue dashed line in Fig. 3a. Figure 3b shows a PL intensity image acquired when the laser focused on one side of the GB. To find out whether the PL intensity distribution is caused by photon scattering effect which indicates the PL distribution caused by excitation light multiple reflection and traveling between crystal and substrate surfaces, we conduct carrier concentration dependent PL distribution measurement. In theory, the spreading of PL should be independent of carrier concentration if it is caused by photon scattering. However, as is shown in Supplementary Fig. 4, the PL distribution is much narrower under high carrier concentration, which is due to a decrease of effective carrier diffusion length when Auger recombination occurs at high carrier concentration. Detailed analysis about the PL intensity mapping method can be found in Supplementary Note 2. And the surface roughness issues have been ruled out by choosing the flat surface with good adherence to the cover glass. And thus, the PL intensity distribution is confirmed to be dominated by carrier density distribution which is determined by diffusion. By superimposing the PL intensity image and the surface photographic image as Fig. 3c, a discontinuous PL intensity was observed, i.e., the PL intensity decreases abruptly on the other side of the GB, indicating that GBs can block at least one type of charger carrier. This effect can be explained by the presence of a band bending at the grain boundaries, as illustrated in Fig. 4c, which blocks one type of charges while extract the other type of charges at the grain boundaries. The band bending at GBs can be induced by self-doping of perovskites due to composition non-uniformity. For example, our previous studies showed that the loss of MAI would convert the material into n-type^43^. GBs loss MA faster than grain interior, which would make GBs area self n-doped and thus forms a band bending. The band-bending at grain boundary area has been frequently observed in Kelvin probe force microscopy study in literatures, and was also observed by us^44^. This result demonstrates this method is effective for imaging the carrier transport, and the regular GBs reflect the carriers.

### Carrier recombination dynamics study at TBs

We then conducted PL mapping measurement on a MAPbI_3_ single crystal sample with TBs as identified in the polarized optical microscope image (Fig. 3d). The corresponding PL imaging shown in Fig. 3e presents a symmetrical intensity distribution with the diameter considerably larger than the focused excitation laser spot. The continuous PL intensity distribution across the TBs indicates that neither electrons nor holes are blocked by the TBs during their transportation, which is consistent with the SPCM measurement result. And the symmetric shape of PL mapping image even crossing the TBs (Fig. 3f) indicates that the carrier could diffuse into all directions with similar velocity. It means that the carrier mobility does not show an obvious dependency on the crystallographic orientation. Note that negligible difference of PL distribution over the twin domains was observed. It could also be explained that the change of crystal orientation is very small in this type of twin boundary. To exclude the impact of samples difference on PL imaging result, PL imaging on a sample that has both a GB and TBs was conducted within one scan, and the result is shown in the Supplementary Movie 1. And the striking difference was also observed even in the same sample.

To study the impact of TBs on carrier recombination kinetics, PL intensity and PL recombination lifetime were mapped based on the single crystals with TBs. As shown in Fig. 4a, the PL intensity shows a distribution with the stripe patterns corresponding to ferroelastic domains, while the variation of PL intensity is less than 5%. We speculate that this PL intensity variation might come from the difference in the absorption of the pump laser, since the laser is also linearly polarized. It should be noted that the TB areas do not have a significantly drop of PL intensity nor PL lifetime (Fig.4b), which suggests that TBs do not behave as additional charge recombination centers in MAPbI_3_ materials.

The striking difference of the TBs and GBs on carrier transport and recombination should originate from their different atomic defect structure. It is well known that GB area in MHP material usually contains large amount of zero-dimensional defects including vacancies, interstitial and substitutional atoms, dangling bonds, and also one-dimensional dislocations defects. The accumulation of defects, especially for defects associated with non-stoichiometric composition like vacancies and dangling bonds, makes perovskite at the GB area non-uniform in composition and thus self-doped. As discussed before and schematically shown in the left panel of Fig. 4c, the self-doped perovskite would cause a band bending at the GB area. And the energy barrier would hinder the transport of at least one type of photo-generated carriers across the GB to the neighboring grain, thus giving rise to the PL plateau within the grain. In addition, the high concentration of defects at the GB area will lead to the non-radiative recombination of the charge carriers, and therefore causes the PL quenching. While for TBs as a special type of two-dimensional defects, their formation in principle does not involve the generation of additional point defects or accumulation of dislocations. As a result, there are neither band bending that impedes the carrier transport nor the trap states that induce the non-radiative carrier recombination, as schematically shown in the right panel of Fig. 4d. And no obvious potential variation was further confirmed with KPFM mapping at the area with obvious TBs as shown in Supplementary Fig. 6. As a result, this give rise to the electronic benign nature of TBs.

## Discussion

In conclusion, we have investigated the influence of TBs on carrier transport in MAPbI_3_ single crystals with multiple analytical methods including SPCM, c-AFM, KPFM, PL imaging, as well as PL intensity and lifetime mapping. It is found that the TBs neither affect the carrier transport across them nor behave as non-radiative recombination centers, in contrast to the regular GBs. The electronic benign nature of the TBs should be related to their unique defect structure, which does not involve the generation of additional point defects or dangling bonds. Our results also reveal that the TBs are easier to be attacked by moisture than grain interior.

## Methods

### Growth of thin single crystals

The MAPbI_3_ thin single crystals were grown by a space confined lateral crystal growth method reported previously^27^. MAPbI_3_ solution in GBL with a concentration of 1.6 M was inserted into two substrates and then placed on a 60 °C hot plate. The temperature was gradually increased to 120 °C for crystal growth. The polarized optical microscopy of the single crystals was performed on an Olympus BX61 microscope in reflection mode with a crossed Nicols configuration. The XRD pattern was measured with a Bruker-AXS D8 Discover Diffractometer.

### Single crystal lateral device fabrication and characterization

The MHP single crystal lateral device was fabricated by depositing 50 nm thick Au electrode on the single crystal surface through a metal mask by thermal evaporation. For the devices on flexible substrates, the single crystals were transferred from the glass substrate to the flexible substrates by pressuring a double-side tape coated polyethylene terephthalate (PEN) substrate on the single crystals and then removing the glass substrate. The SPCM measurement was conducted by fixing the single crystal device on a computer-controlled X-Y scanning stage (PI Q-545.240) with scanning step size of 1 μm. A laser diode with the wavelength of 808 nm was used as the excitation source, modulated by a mechanical chopper at the frequency of 35 Hz, and focused through a ×50 microscope objective onto the device. The external bias was applied to the device with a Keithley 2400 source meter, and photocurrent signal at each beam position was recorded by a lock-in amplifier (Stanford Research Systems SRS 830).

### PL imaging and confocal PL and TRPL mapping measurement

For spatial resolved PL distribution measurement, single crystal sample is excited with CW 532 nm laser (Coherent Verdi) focused with an objective (Nikon CFI Plan Apo Lambda ×60 0.95 NA). PL signal is collected by the same objective and read out with camera (Princeton Instruments, Pixis 1024B). In the confocal PL intensity and lifetime measurements, samples are excited with a 408 nm pulsed laser (MDL 300, PicoQuant) at 40 μm cm^−2^ pulse energy density (pulse width 180 ps). The PL signal is detected by a single-photon avalanche photodiode (τ-SPAD, PicoQuant), and recorded with a time-correlated single-photon counting system (PicoHarp 300, PicoQuant). As is shown in Supplementary Fig. 5, the PL intensity went through a fast decay from 0 to 15 ns, and a slow decay after 15 ns. The initial fast drop could be due to two main reasons, bimolecular carrier recombination and carrier diffuse out of the collection area. However, we want to understand the trap-carrier interaction at the TBs, so we focus on the PL intensity decay after 15 ns, which can be well fitted with a single exponential decay model. And no obvious lifetime change can be observed associated with the TBs.

## Supplementary information

Supplementary Information

Description of Additional Supplementary Files

Supplementary Movie 1

## Data Availability

The data that support this study are available from the corresponding author upon reasonable request.

## Source data

Source Data
