# Supplementary material for: Benign ferroelastic twin boundaries in halide perovskites for charge carrier transport and recombination
Source: Nat Commun. 2020 May 5;11:2215. doi: 10.1038/s41467-020-16075-1 (PMC7200693; doi:10.1038/s41467-020-16075-1)
Supplement: Supplementary file 1 — Supplementary Information [file 41467_2020_16075_MOESM1_ESM.pdf]

Supplementary Information for

Benign Ferroelastic Twin Boundaries in Halide Perovskites for Charge Carrier Transport and Recombination

Xun Xiao[1#], Wenhao Li[2#], Yanjun Fang[3#], Ye Liu[3], Yuchuan Shao[1], Shuang Yang[3], Jingjing Zhao[3], Xuezeng Dai[1] Rashid Zia[2], and Jinsong Huang[1,3*]

[1] *Department of Applied Physical Sciences, University of North Carolina, Chapel Hill, NC 27599, USA.*

[2] *School of Engineering and Department of Physics, Brown University, Providence, Rhode Island 02912, USA*

[3] *Department of Mechanical and Materials Engineering and Nebraska Center for Materials and Nanoscience, University of Nebraska-Lincoln, Lincoln, NE 68588, USA.*

[#]X. X., W. L., and Y. F. contributed equally to this work. [*] Correspondence to JH, Email: jhuang@unc.edu

(a)

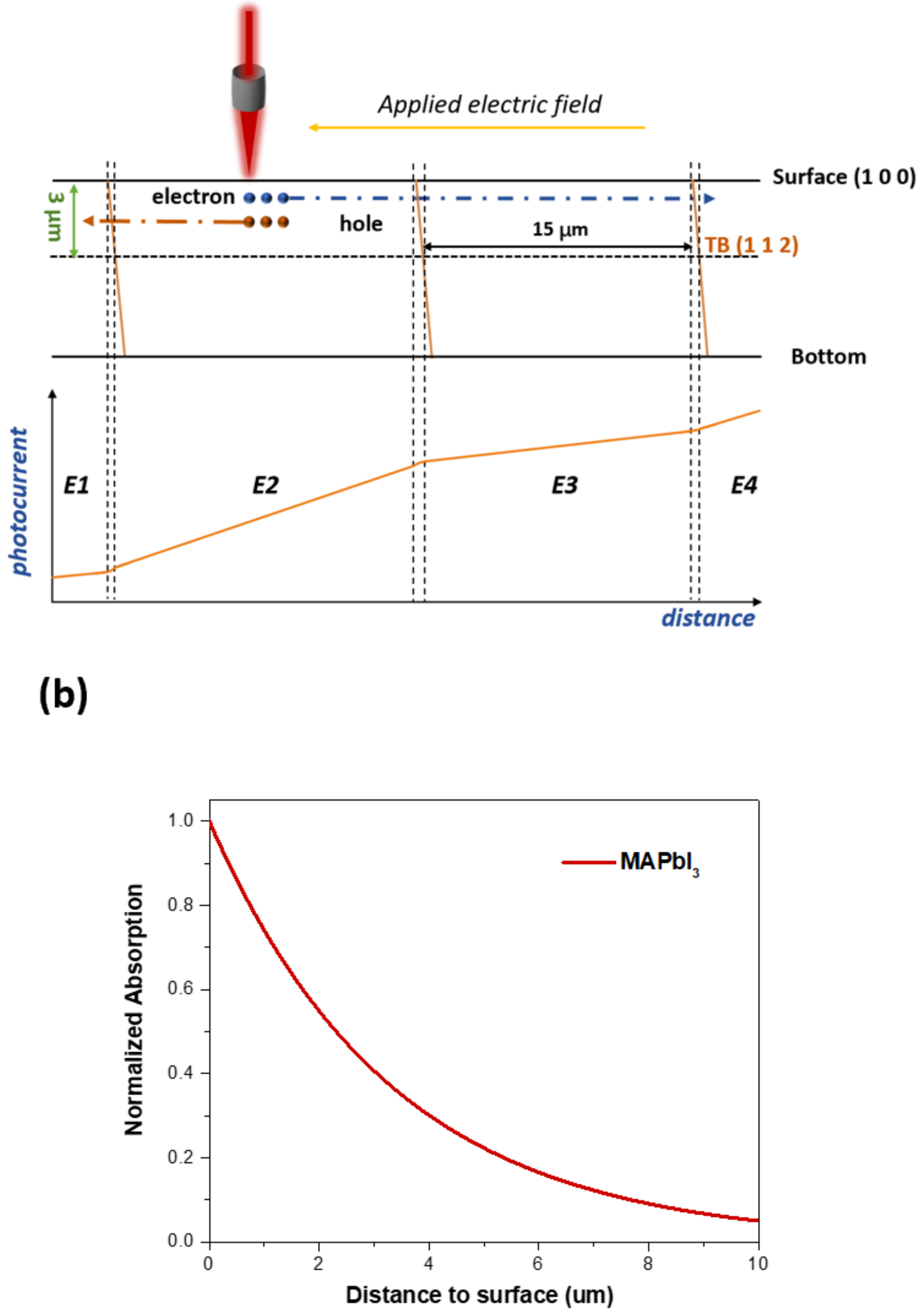

Supplementary Figures:

Supplementary Figure 1: Cross-section view of Scanning photocurrent mapping (a) Scheme of photocurrent mapping to resolve electric field distribution in domains if there is electric field discontinuity **(b)** Normalized absorbed light intensity of 808 nm laser with respect to distance to surface.

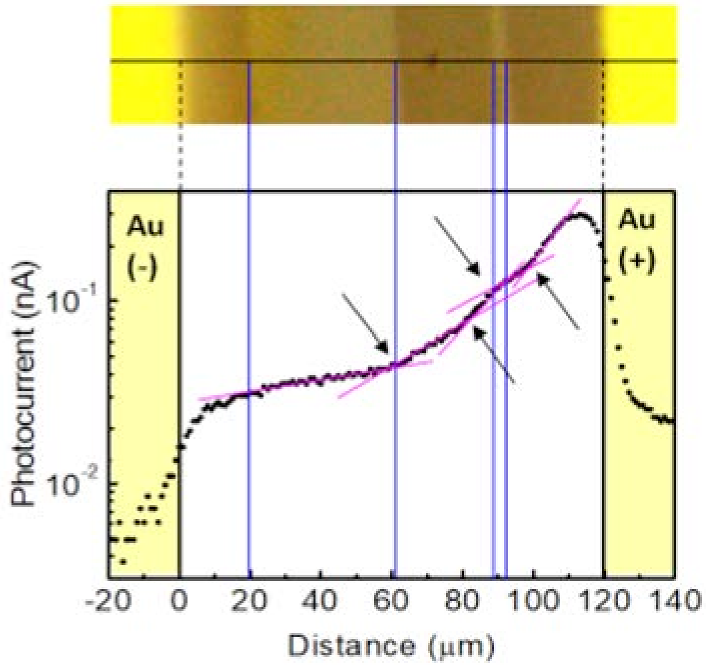

Supplementary Figure 2: SPCM of un-passivated sample. Polarized optical image of the $MAPbI_3$ single crystal lateral device (upper panel) and the typical photocurrent line scan profile of the un-passivated crystal (lower panel) with the external bias of 5 V mm^{-1}.

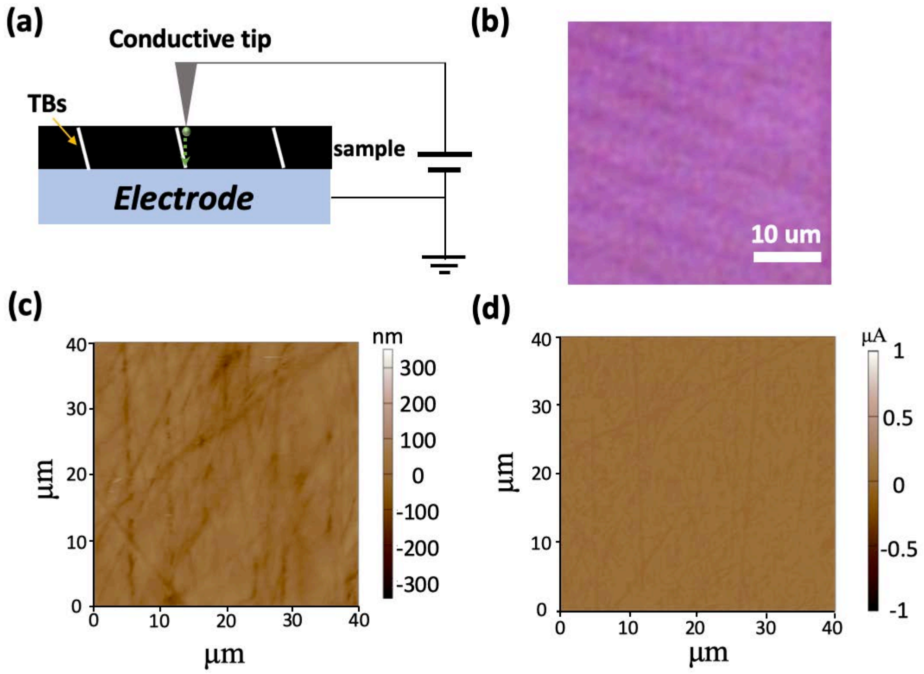

Supplementary Figure 3: c-AFM on TB area. **(a)** c-AFM measurement setup, **(b)** polarized light image of the c-AFM mapping area, **(c)** Height and **(d)** current image of c-AFM measurement.

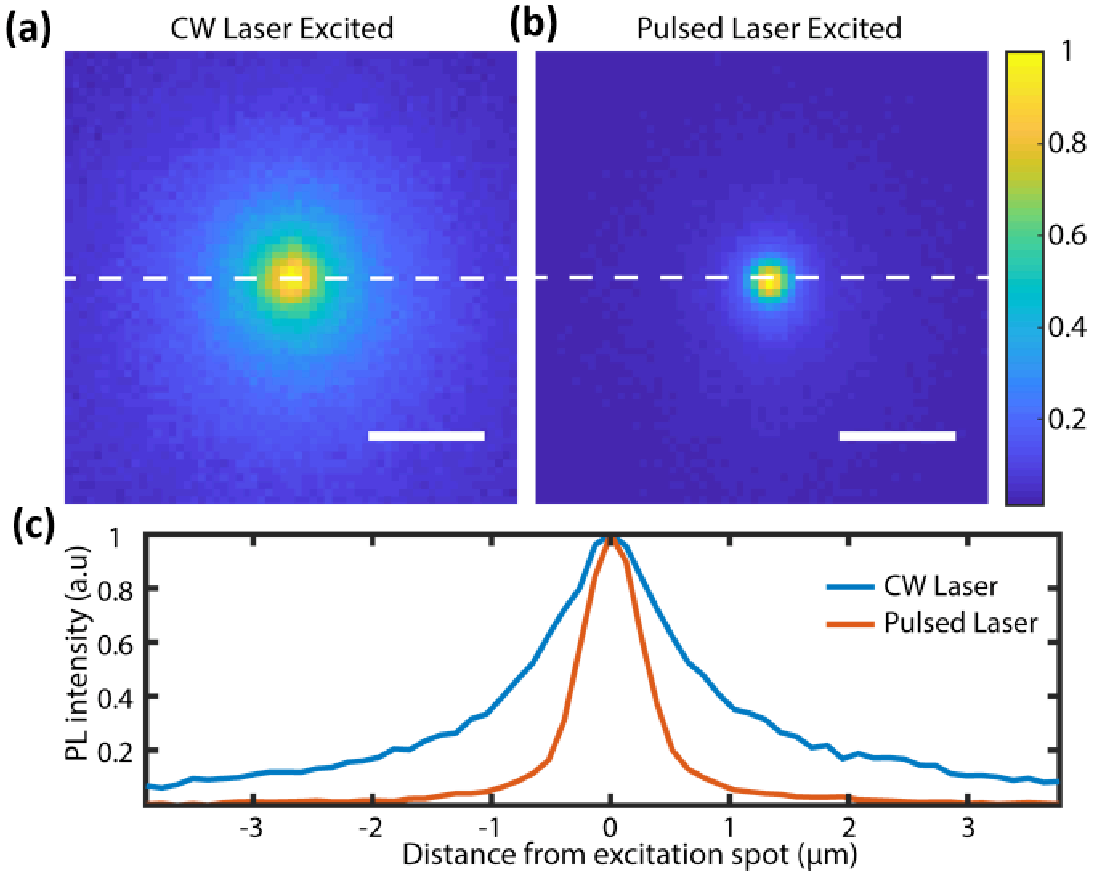

Supplementary Figure 4: CW and Pulsed laser based PL imaging. (a) PL imaging under CW laser excitation; **(b)** PL imaging under pulsed laser excitation; **(c)** PL intensity lateral distribution corresponding to the white dashed line in **(a)** & **(b)**. Scale bars are 2 μm.

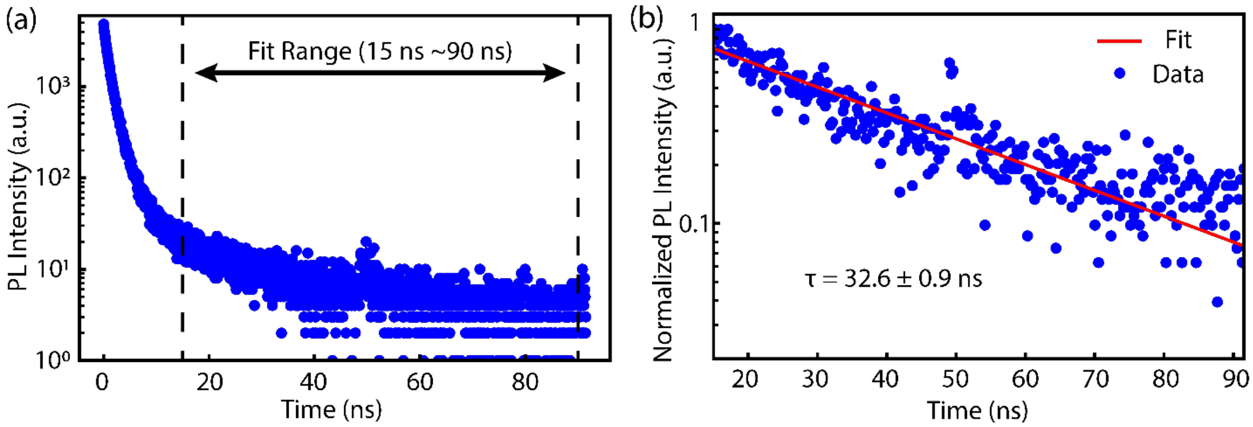

Supplementary Figure 5: Example of PL decay in TRPL mapping. Time resolved PL intensity data and the single exponential fitting on 15 ns to 90 ns time range.

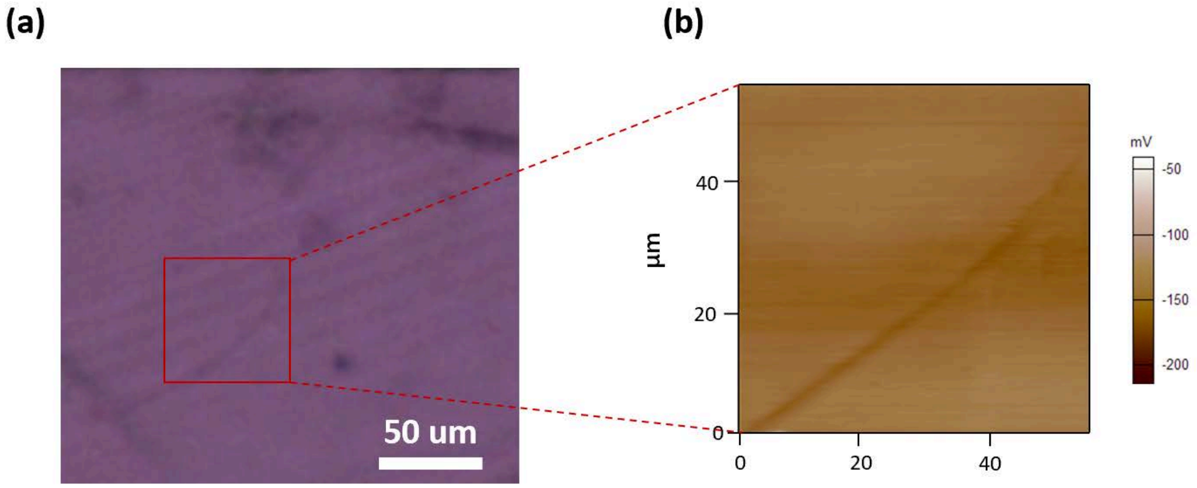

Supplementary Figure 6: KPFM on TB area. **(a)** polarized light image of $MAPbI_3$ single crystal with TBs, red rectangular indicates the KPFM scanning area; **(b)** KPFM scanning result with the selected area.

Supplementary Note 1. Modeling of the impact of electric field fluctuation on photocurrent profile of $MAPbI_3$ single crystal lateral device

Based on the Hecht equation,[1] if the total amount of electron-hole pairs generated at the position x is N_0, then the electrons collected at the anode after a drift time of t_e ($Q_e(t)$)is:

$$Q_e(t) = \frac{q\mu_e}{d}\int_0^{t_e} N_0 e^{-\frac{t'}{\tau}} E(x(t'))\mathrm{d}t' \quad (1)$$

where q is the elementary charge, d is the conductive channel width, μ_e is the electron mobility, τ is the carrier recombination lifetime, $x(t)$ is the position of the electrons after the drift time of t, and $E(x(t))$ is the electric field intensity at the position $x(t)$. Similarly, the holes collected at the cathode after a drift time of t_h ($Q_h(t)$) is:

$$Q_h(t) = \frac{q\mu_h}{d}\int_0^{t_h} N_0 e^{-\frac{t'}{\tau}} E(t')\mathrm{d}t' \quad (2)$$

where μ_h is the hole mobility. The photocurrent (I) includes the contribution from both electrons and holes, which can be derived by replacing N_0 in Supplementary Equation (1) and (2) with charge generation rate and sum them together:

$$I = \frac{\eta q P}{h\nu d}\left(\mu_e \int_0^{t_e} e^{-\frac{t'}{\tau}} E(x_e(t'))\mathrm{d}t' + \mu_h \int_0^{t_h} e^{-\frac{t'}{\tau}} E(x_h(t'))\mathrm{d}t'\right) \quad (3)$$

where η is the external quantum efficiency, P is the incident light intensity, h is the Planck constant, and ν is the incident light frequency. It is evident that the charge transit time to the electrode depends on the electric field distribution $E(x(t))$ as well as the light excitation position x in the conductive channel. With the proposed electric field distribution non-uniformity shown in Supplementary Figure 1a, the electron transit time is:

$$t_{\mathrm{e}} = \begin{cases} \frac{\frac{d}{4}-x}{\mu_{\mathrm{e}}E_1} + \frac{\frac{d}{4}}{\mu_{\mathrm{e}}E_2} + \frac{\frac{d}{4}}{\mu_{\mathrm{e}}E_3} + \frac{\frac{d}{4}}{\mu_{\mathrm{e}}E_1}, & 0 < x < \frac{d}{4} \\ \frac{\frac{d}{2}-x}{\mu_{\mathrm{e}}E_2} + \frac{\frac{d}{4}}{\mu_{\mathrm{e}}E_3} + \frac{\frac{d}{4}}{\mu_{\mathrm{e}}E_1}, & \frac{d}{4} \le x < \frac{d}{2} \\ \frac{\frac{3d}{4}-x}{\mu_{\mathrm{e}}E_3} + \frac{\frac{d}{4}}{\mu_{\mathrm{e}}E_1}, & \frac{d}{2} \le x < \frac{3d}{4} \\ \frac{d-x}{\mu_{\mathrm{e}}E_1}, & \frac{3d}{4} \le x < d \end{cases} \tag{4}$$

Similarly, the hole transit time is:

$$t_{\mathrm{h}} = \begin{cases} \frac{x}{\mu_{\mathrm{h}}E_1}, & 0 < x < \frac{d}{4} \\ \frac{x-\frac{d}{4}}{\mu_{\mathrm{h}}E_2} + \frac{\frac{d}{4}}{\mu_{\mathrm{h}}E_1}, & \frac{d}{4} \le x < \frac{d}{2} \\ \frac{x-\frac{d}{2}}{\mu_{\mathrm{h}}E_3} + \frac{\frac{d}{4}}{\mu_{\mathrm{h}}E_2} + \frac{\frac{d}{4}}{\mu_{\mathrm{h}}E_1}, & \frac{d}{2} \le x < \frac{3d}{4} \\ \frac{x-\frac{3d}{4}}{\mu_{\mathrm{h}}E_1} + \frac{\frac{d}{4}}{\mu_{\mathrm{h}}E_3} + \frac{\frac{d}{4}}{\mu_{\mathrm{h}}E_2} + \frac{\frac{d}{4}}{\mu_{\mathrm{h}}E_1} & \frac{3d}{4} \le x < d \end{cases} \tag{5}$$

By substituting Supplementary Equation (4) and (5) into Supplementary Equation (3) and do the integration, we can derive the photocurrent profile in the conductive channel of the lateral device as shown in Supplementary Figure 1b, supposing $\mu_{\mathrm{e}}\tau = 1\times10^{-4}$ cm^2 V^{-1}, $\mu_{\mathrm{h}}\tau = 3\times10^{-4}$ cm^2 V^{-1}, $E_1 = 5000$ V m^{-1} (0.5V across 100 μm channel), $E_2 = 2000$ V m^{-1}, $E_3 = 8000$ Vm^{-1}, and $d = 100$ μm.

Supplementary Note 2. Details of PL imaging and PL lifetime mapping data.

To avoid surface roughness related artifacts, flat sample with good adhesion to the cover glass was chosen to conduct the PL imaging measurement. And to ensure the wide PL intensity distribution is not due to photon scattering effect, the PL imaging measurement is also performed with a 408 nm pulse laser with much stronger instantaneous power as pumping light source. If the expansion of the PL is due to light scattering, the resulting PL distribution should not change since the surface roughness and PL wavelength is still the same. However, as is shown in Supplementary Figure 4, the PL intensity expansion is narrower compared to the case of the CW laser excitation. This effect could be explained by the effective diffusion length decrease introduce by the high energy density laser excitation. The diffusion length $L_D = \sqrt{D\tau}$, D is the diffusivity, which is an intrinsic property of the material, and τ is the carrier recombination lifetime. τ decrease might be the result of Auger recombination at high carrier concentration, which leads to a decrease in the diffusion length.

Reference

1. Ruzin, A. Nemirovsky, Y., Statistical models for charge collection efficiency and variance in semiconductor spectrometers. *J. Appl. Phys.* **82**, 2754-2758 (1997).
