# Supplementary material for: Benign ferroelastic twin boundaries in halide perovskites for charge carrier transport and recombination
Source: Nat Commun. 2020 May 5;11:2215. doi: 10.1038/s41467-020-16075-1 (PMC7200693; doi:10.1038/s41467-020-16075-1)
Supplement: Supplementary file 2 — Description of Additional Supplementary Files [file 41467_2020_16075_MOESM2_ESM.pdf]

Description of Additional Supplementary Files

Supplementary Movie 1: In-situ PL imaging of GB and TBs.
